# Paracrine and autocrine regulation of gene expression by Wnt-inhibitor Dickkopf in wild-type and mutant hepatocytes

**DOI:** 10.1186/s12918-017-0470-9

**Published:** 2017-10-13

**Authors:** Niklas Hartung, Uwe Benary, Jana Wolf, Bente Kofahl

**Affiliations:** 1 University of Potsdam, Institute of Mathematics, Karl-Liebknecht-Str. 24, Potsdam, 14476 Germany; 2 Mathematical Modelling of Cellular Processes, Max Delbrueck Center for Molecular Medicine, Robert-Roessle-Str. 10, Berlin, 13125 Germany; 3 Current address: Institute of Physics, University of Freiburg, Hermann-Herder-Str. 3, Freiburg i. Br., 79104 Germany

**Keywords:** Wnt/ *β*-catenin signalling pathway, Dickkopf diffusion and feedback regulation, APC concentration gradient, Mathematical model, Paracrine and autocrine regulation, Reaction-diffusion system

## Abstract

**Background:**

Cells are able to communicate and coordinate their function within tissues via secreted factors. Aberrant secretion by cancer cells can modulate this intercellular communication, in particular in highly organised tissues such as the liver. Hepatocytes, the major cell type of the liver, secrete Dickkopf (Dkk), which inhibits Wnt/ *β*-catenin signalling in an autocrine and paracrine manner. Consequently, Dkk modulates the expression of Wnt/ *β*-catenin target genes. We present a mathematical model that describes the autocrine and paracrine regulation of hepatic gene expression by Dkk under wild-type conditions as well as in the presence of mutant cells.

**Results:**

Our spatial model describes the competition of Dkk and Wnt at receptor level, intra-cellular Wnt/ *β*-catenin signalling, and the regulation of target gene expression for 21 individual hepatocytes. Autocrine and paracrine regulation is mediated through a feedback mechanism via Dkk and Dkk diffusion along the porto-central axis. Along this axis an APC concentration gradient is modelled as experimentally detected in liver. Simulations of mutant cells demonstrate that already a single mutant cell increases overall Dkk concentration. The influence of the mutant cell on gene expression of surrounding wild-type hepatocytes is limited in magnitude and restricted to hepatocytes in close proximity. To explore the underlying molecular mechanisms, we perform a comprehensive analysis of the model parameters such as diffusion coefficient, mutation strength and feedback strength.

**Conclusions:**

Our simulations show that Dkk concentration is elevated in the presence of a mutant cell. However, the impact of these elevated Dkk levels on wild-type hepatocytes is confined in space and magnitude. The combination of inter- and intracellular processes, such as Dkk feedback, diffusion and Wnt/ *β*-catenin signal transduction, allow wild-type hepatocytes to largely maintain their gene expression.

**Electronic supplementary material:**

The online version of this article (doi:10.1186/s12918-017-0470-9) contains supplementary material, which is available to authorized users.

## Background

Cells are exposed to numerous external factors, arriving from environmental sources as well as surrounding cells. Factors secreted by cells into the extracellular space can be used for intercellular communication and by that for the coordination of cellular functions on the cell population or tissue level. Cancer cells also secrete factors but the composition and amount can differ compared to that of wild-type cells. Thereby, cancer cells can alter their microenvironment and exert a different impact on surrounding cells than wild-type cells. In consequence, gene expression and cellular functions of the surrounding cells can change. The effects are of critical importance in tissues with a complex spatial specialisation of cells, as e.g. in the liver. Here, we investigate how Dickkopf (Dkk), a factor secreted by normal and mutated hepatocytes and acting in an autocrine as well as paracrine manner, influences gene expression in the hepatic cellular environment.

**Fig. 1:**
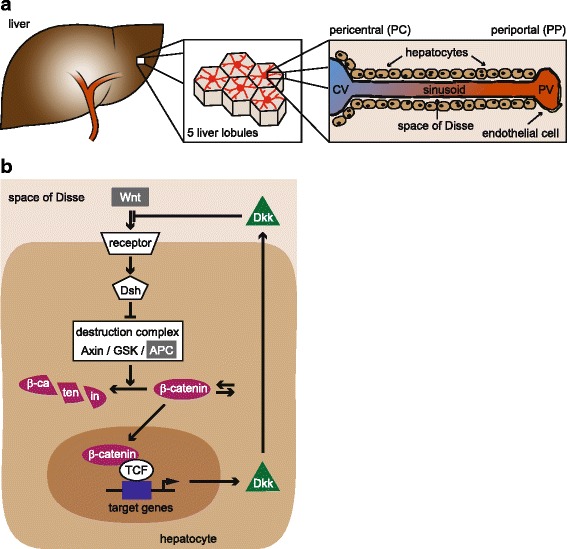
Simplified scheme of liver architecture and Wnt/ *β*-catenin signalling. **a:** The human liver is composed of two lobes of unequal size. It is supplied with blood from two sources, the hepatic portal vein and the hepatic arteries. The blood is distributed into capillaries that enter the liver lobules, which are hexagonal substructures that form the functional units of the liver. The blood flows through the sinusoids of the lobules from the portal vein (PV) to the central vein (CV). Hepatocytes surround the sinusoids separated by space of Disse. Typically, 15 to 25 hepatocytes align along the porto-central axis. **b:** The central component of the Wnt/ *β*-catenin signalling pathway is *β*-catenin (purple). *β*-Catenin is constantly produced and degraded and maintains a low expression level. The degradation is predominantly mediated by a destruction complex comprising APC (grey), Axin and the kinase GSK. Upon stimulation by Wnt ligands (grey), the Wnt receptor complex activates intracellular proteins including Dishevelled (Dsh) that induce a partial inactivation of the destruction complex. In consequence, *β*-catenin degradation is impaired and more *β*-catenin can translocate into the nucleus. There, it binds to transcriptional regulators of the TCF family and co-regulates the expression of target genes. In particular, *β*-catenin/TCF complexes induce the expression of Dkk (green). Dkk is secreted by the cells and acts as an inhibitor of the pathway by competing with Wnt for receptor binding. The colours highlighting Wnt, APC, *β*-catenin, target genes and Dkk correspond to the colour code used throughout the manuscript

The liver is the largest gland of the mammalian body with a multitude of functions including blood detoxification, protein synthesis, regulation of glucose metabolism, and production of hormones and bile. It has long been known that hepatocytes, the predominant cell type of the liver, accomplish distinct biochemical tasks dependent on their location along the periportal (PP) - pericentral (PC) axis of the sinusoids of liver lobules (Fig. 1 a) [1–7]. For example, glycolysis and glutamine synthesis occur predominantly in the PC region, while gluconeogenesis and urea formation occur predominantly in the PP region [2, 3, 5, 7, 8]. This physiological phenomenon is referred to as functional zonation and is paralleled by distinct gene expression programmes [1–7]. The underlying mechanisms causing functional zonation are intensively studied. According to current hypotheses extracellular factors, such as oxygen, hormones, or morphogens, are differently abundant along the porto-central axis and control the particular gene expression of hepatocytes located in specific zones of the liver sinusoids [7, 8].

Experimental findings indicate that intracellular components of the Wnt/ *β*-catenin signalling pathway, in particular *β*-catenin and adenomatous polyposis coli (APC), are also important contributors to functional zonation [9–12]. Immunostaining in liver sections showed that the total intracellular APC concentration decreases from one hepatocyte to the next along the porto-central axis, i.e. a gradient of APC exists along the sinusoid [10]. This gradient is paralleled by differential expression of Wnt/ *β*-catenin target genes associated with ammonium metabolism such as glutamine synthetase [10]. Other immunohistochemical experiments revealed that loss of *β*-catenin or loss of Wnt co-receptor low-density-lipoprotein receptor-related proteins 5 and 6 (LRP) impair zonation emphasising the involvement of Wnt/ *β*-catenin signalling in its proper formation [9, 11, 12].

The central component of the Wnt/ *β*-catenin signalling pathway is the transcriptional regulator *β*-catenin (Fig. 1 b). *β*-Catenin associates with transcription factors of the T-cell factor/lymphoid enhancer factor (TCF) family in the nucleus to regulate the expression of specific target genes [13–15]. The abundance of *β*-catenin is controlled by a multi-protein complex, referred to as destruction complex, in which APC, Axin, and glycogen synthase kinase 3 (GSK3) are central components. Bound to APC and Axin in this destruction complex, *β*-catenin becomes phosphorylated by GSK3 resulting in the proteasomal degradation of *β*-catenin [16–18]. This process maintains low concentrations of *β*-catenin in the cell and thus prevents the activation of Wnt/ *β*-catenin-dependent target genes.

Extracellular stimulation of a cell with the morphogen Wnt directs the transmembrane receptor Frizzled (Frz) and co-receptor LRP into close proximity establishing the Wnt/Frz/LRP complex [19, 20]. This formation of a receptor complex allows signal transduction to downstream proteins in the cell resulting in the partial inhibition of the destruction complex [19, 21]. The exact molecular mechanism has yet to be resolved but Dishevelled (Dsh) seems to play a central role [19, 21, 22]. The partial inhibition of the destruction complex impairs *β*-catenin degradation [23, 24]. In consequence, more *β*-catenin can enter the nucleus, associate with TCF transcription factors, and regulate target gene expression [13–15].

Various extracellular proteins are known to modulate Wnt/ *β*-catenin signal transduction on the receptor level such as secreted frizzled-related proteins or Dkk proteins [25–27]. In vertebrates, the Dkk protein family comprises four members (Dkk1, Dkk2, Dkk3 and Dkk4) that have different biochemical and physiological properties [25, 27]. In our study, Dkk1 is exclusively considered (hereafter referred to as Dkk). Secreted Dkk binds extracellularly to LRP and thereby interferes with the formation of the Wnt/Frz/LRP complex [28–30]. By that mechanism, Dkk can affect downstream signalling events and hence target gene expression. Dkk expression is induced by Wnt/ *β*-catenin signalling establishing an autocrine feedback loop [31, 32]. In addition, secreted Dkk has been proposed to diffuse and influence adjacent cells in a paracrine manner [33, 34].

In hepatocellular carcinoma (HCC), a prominent type of liver cancer, Dkk is overexpressed [35, 36]. The secreted protein can readily be monitored in the blood and is therefore discussed as potential biomarker for the diagnostics of HCC cells in clinical research [35, 37, 38]. HCC cells frequently harbour mutations in *β*-catenin (19-33%) [39–42], Axin (5-15%) [40, 41, 43] or APC (2%) [41]. These mutations can alter the destruction complex-dependent degradation of *β*-catenin resulting in *β*-catenin accumulation even in the absence of an external Wnt stimulus. Nuclear accumulation of *β*-catenin has been detected in HCC samples [38, 42, 44] and may be the underlying reason for Dkk overexpression.

In the present study, we use a mathematical modelling approach to investigate the autocrine and paracrine effects of Dkk on the mRNA expression of target genes that are regulated by Wnt/ *β*-catenin signalling in the liver. Previously, various mathematical models have been published [45–47] investigating different aspects of the Wnt/ *β*-catenin signalling pathway, such as receptor activation [48–50], regulation of signal transduction [49, 51–57], feedback mechanisms [58–60], interplay of *β*-catenin with E-cadherin [55, 61] or the control of hepatic target gene expression [54, 62, 63]. Modelling efforts addressed spatial aspects within tissues especially in the crypts of the colon [64–67]. Also the impact of diffusion of pathway activators and inhibitors and/or crosstalk with other signalling pathways has been investigated [34, 68–75]. Our reaction-diffusion model combines many of these diverse aspects by integrating Dkk-regulated events at the Wnt receptor level with signal transduction processes and gene regulation in the context of a hepatic APC concentration gradient. It is considered that Dkk exerts an autocrine influence because produced and secreted Dkk can act back on the LRP receptor of the secreting cell itself. Furthermore, Dkk mediates a paracrine regulation: secreted Dkk diffuses and binds the LRP receptor of adjacent cells. Our investigation is set in a liver-specific context in which a concentration gradient of APC along the porto-central axis exists.

In addition, we explore how a single mutant cell may affect gene expression in adjacent wild-type hepatocytes. We simulate this single mutant cell by a reduction of the destruction-complex-dependent degradation of *β*-catenin. This reduction reflects the aberrant regulation caused by APC, Axin or *β*-catenin mutations that have been associated with HCC [39–43]. The analysis of the model without a mutant cell reveals that Dkk feedback and diffusion have opposite effects on target gene expression along the porto-central axis. We demonstrate that already a single mutant cell considerably increases overall Dkk concentration compared to the wild-type scenario. This mutant cell-derived Dkk can diffuse along the porto-central axis, affect signalling in adjacent wild-type cells, and reduce their target gene mRNA expression. However, the elevated Dkk levels have a limited impact on gene expression of wild-type hepatocytes, which, moreover, is confined to the immediate vicinity of the mutant cell.

## Methods

The spatial model of Dkk-regulated Wnt signalling consists of a set of ordinary differential equations (ODEs) for each cell. Cells were coupled by a reaction-diffusion equation describing the evolution of Dkk, which was discretised by finite differences, yielding a set of ODEs. The equations are provided in Additional file 1 (Section A). Calculations were done with Matlab R2016b (The MathWorks, Natick, MA). Steady state solutions were numerically obtained (stiff ODE solver). The Matlab code is available in Additional file 2.

## Results

### Spatial model of hepatic Wnt signalling considering Dkk feedback and diffusion

#### Structure and components of the model

**Fig. 2:**
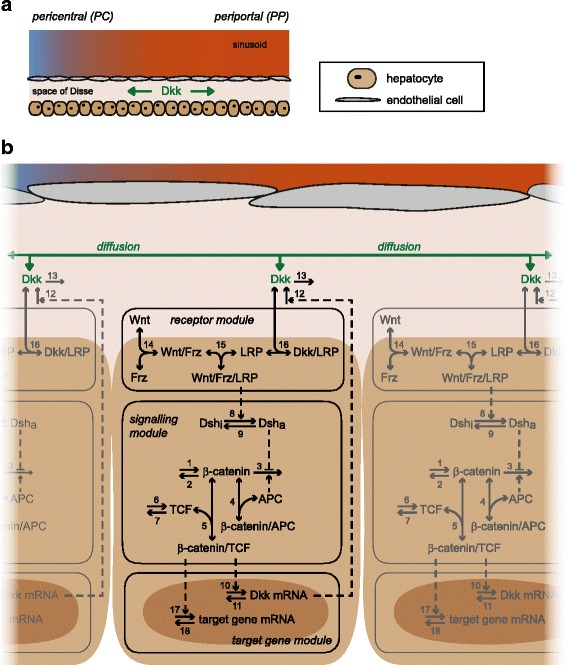
Schematic representation of the mathematical model. **a:** The spatial model considers a single row of 21 adjacent hepatocytes. Each hepatocyte harbours the identical structure of the Wnt/ *β*-catenin signalling pathway shown in (**b**), but differs in its total APC concentration. Dkk may diffuse along the row of cells through the space of Disse as indicated by the green arrows. **b:** Model scheme exemplarily shown for neighbouring hepatocytes. The hepatocytes and their nuclei are indicated by light and dark brown backgrounds, respectively. The reaction scheme is identical in each of the 21 cells. The model is subdivided into three modules: the receptor module, the signalling module, and the target gene module. Components in a complex are separated by slashes. One-headed arrows denote reactions taking place in the indicated direction; double-headed arrows illustrate reversible binding reactions. Dashed arrows represent activation mechanisms; the dashed line ending in T-shape denotes inhibition. The number next to an arrow specifies the number of the reaction. Model equations and the reference parameter set are provided in the Additional file 1 (Section A). Note that Dkk can diffuse (green arrow) to all neighbouring hepatocytes on the right and left side

We aim to investigate the impact of Dkk on target gene expression in the context of an APC gradient in the liver. We focus our modelling approach on the 15-25 hepatocytes that typically align along the porto-central axis parallel to a liver sinusoid (Fig. 1 a) [6]. Our model considers 21 cells that each harbour the identical structure of the Wnt/ *β*-catenin signalling pathway including the regulatory Dkk feedback (Fig. 2) but differ in their total concentration of APC to simulate an APC gradient increasing from PC to PP [10]. Each cell produces Dkk that can influence the Wnt/ *β*-catenin pathway of its producing cell (autocrine feedback) or can diffuse along the portal-central axis and impact the signalling of other cells (paracrine regulation). In our modelling approach, Dkk is the only component that diffuses.

To describe the Wnt/ *β*-catenin signalling pathway, we employ the signal transduction module of a published model [54]. This signalling module focusses on the intracellular interactions of the central signal mediator *β*-catenin (Fig. 2 b, reactions 1-9). *β*-Catenin is produced (reaction 1) and degraded in an APC-independent (reaction 2) and APC-dependent manner (reaction 3). The APC-dependent reaction represents the degradation of *β*-catenin that is mediated by the destruction complex. This reaction can be inhibited by activated Dsh (Dsh _*a*_). Dsh _*a*_ is produced from inactive Dsh (Dsh _*i*_) in a Wnt/Frz/LRP receptor complex-dependent way (reaction 8) and may reconvert into Dsh _*i*_ again (reaction 9). *β*-Catenin can reversibly form a complex with APC (reaction 4). This complex represents the binding of *β*-catenin to APC independent of the destruction complex [51]. In case of high concentrations of *β*-catenin, this complex formation acts as a positive feedback by reducing the concentration of the destruction complex [76]. *β*-Catenin can also reversibly form a complex with the transcription factor TCF (reaction 5). In the model, the resulting *β*-catenin/TCF complex is considered to be the transcriptional activator of gene expression. TCF is produced (reaction 6) and degraded (reaction 7). The pathway components Dsh and APC obey conservation relations; that is, their respective total concentrations remain constant over time (Additional file 1, Section A).

We extend this signalling module by a module of target gene expression (Fig. 2 b, reactions 10-13 and 17-18). In this module, the *β*-catenin/TCF complex activates the transcription of an unspecified target gene mRNA (reaction 17), which we consider as the readout in our model analyses. This unspecified target gene mRNA is degraded (reaction 18) but not translated for the sake of simplicity of the model. In addition to the unspecified target gene mRNA, we include the transcriptional activation of Dkk mRNA by the *β*-catenin/TCF complex (reaction 10). Dkk mRNA is degraded (reactions 11) or translated into Dkk. We do not explicitly consider intracellular Dkk in our model. Rather the two processes of intracellular translation of Dkk mRNA and secretion of Dkk into extracellular space are lumped into one process (reaction 12), which produces extracellular Dkk. The loss of extracellular Dkk (reaction 13) can similarly be understood as a lumped process combining clearance of Dkk from extracellular space and its intracellular degradation.

The model furthermore integrates a previously published receptor module [49], which describes the molecular mechanisms of extracellular Dkk and Wnt binding to the membrane-bound Wnt receptor subunits LRP and Frz (Fig. 2 b). Wnt binds sequentially and reversibly to Frz and LRP, which results in the formation of the Wnt/Frz/LRP complex (reactions 14 and 15, respectively). This complex mediates the activation of Dsh _*i*_ and therefore activates the downstream signalling pathway. The formation of the Wnt/Frz/LRP complex can be inhibited by Dkk. Dkk sequesters unbound LRP into Dkk/LRP complexes (reaction 16), that do not mediate Dsh _*i*_ activation. The total concentrations of LRP and Frz are conserved in our model.

The model focusses on spatial effects along the porto-central axis, which is discretised into 21 segments of equal size representing the 21 hepatocytes being typically located along the axis [6]. In the model, Dkk can diffuse and therefore it can act on LRP of the Dkk producing cell as well as on LRP of neighbouring cells (Fig. 2). Dkk secreted by hepatocytes is presumed to diffuse in extracellular space (space of Disse) to neighbouring cells rather than being transported by blood flow. Consequently, a convection term of Dkk is not considered here. In our model, only 1-D diffusion along the porto-central axis is considered. Wnt is also an extracellular component but in contrast to Dkk, a Wnt diffusion is not considered in the model. To our knowledge, there exists no experimental evidence showing diffusion of Wnt in the liver. In the following paragraphs, we will investigate the consequences of different hypothesised concentration gradients of Wnt. These different concentration gradients are explicitly defined by setting total Wnt for each of the 21 cells to a particular value.

Model parameters are collected from several experimental [77–80] and theoretical publications [49, 51, 54, 60]. Synthesis processes that are not regulated are described by constant rates; all other reactions follow mass-action kinetics. The inhibitory impact of Dsh _*a*_ on APC-dependent *β*-catenin degradation is implemented as an inhibitory function as commonly used in mathematical models of signal transduction pathways [81]. The chosen kinetics represent the simplest possible descriptions of reactions for which the detailed mechanistics of the individual steps are missing and which are widely used in models of signal transduction pathways [81–84]. Details on the model parameters and model equations are provided in the Additional file 1 (Section A). We analyse the system in steady-state. Uniqueness of the steady-state is shown in the Additional file 1 (Section B).

#### Effect of the autocrine and paracrine regulation by Dkk on the target gene mRNA expression in hepatocytes

**Fig. 3:**
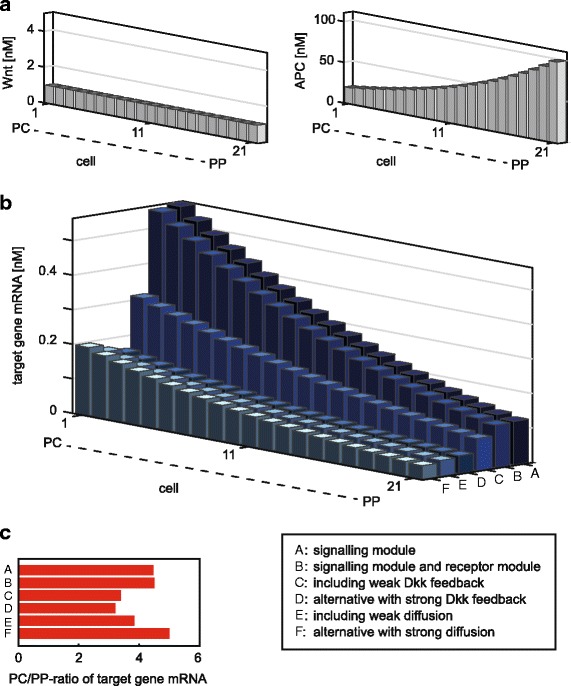
Effect of selected model processes on target gene mRNA expression along the porto-central axis. **a:** A constant total Wnt concentration for each cell (1 nM) and an exponential gradient of total APC concentration increasing 5-fold from PC (20 nM) to PP (100 nM) is assumed. **b:** The target gene mRNA expression profiles are shown for different model setups: **A:** The expression profile is shown if only the signalling module is considered (Wnt/Frz/LRP complex concentration is set to 1 nM, reaction rates *k*_10_ to *k*_16_ are set to 0; this setup is equivalent to the published signalling model [54]). This model setup is successively extended by consideration of **B:** the receptor module without Dkk feedback and diffusion, **C:** the receptor module with a weak Dkk feedback (*k*_12_=2·10^−3^ min ^−1^) and no diffusion, **D:** the receptor module with a stronger Dkk feedback (*k*_12_=2·10^−2^ min ^−1^) and no diffusion, **E:** the receptor module with a stronger Dkk feedback *k*_12_=2·10^−2^ min ^−1^) and weak diffusion (*D*=10 $\frac {\mu m^{2}}{s}$), **F:** the receptor module with a stronger Dkk feedback *k*_12_=2·10^−2^ min ^−1^) and stronger diffusion (*D*=500 $\frac {\mu m^{2}}{s}$). **c:** The PC/PP target gene mRNA expression ratios for the model setups presented in (**b**) are compared

Autocrine and paracrine regulations are introduced in the model via Dkk feedback and Dkk diffusion. To study their effects we analyse the target gene mRNA expression in cells along the porto-central axis. Histochemical experiments in liver sections revealed that hepatic target gene expression is associated with an APC concentration gradient that increases from PC to PP region [10]. Moreover, while Wnt ligands have to be present [9, 10], to our knowledge no experimental evidence for gradient in these ligands exists. We therefore start our model analysis assuming an APC gradient that increases 5-fold from 20 nM (PC) to 100 nM (PP) and equal total Wnt concentration of 1 nM acting upon the individual hepatocytes (Fig. 3a). The choice of the maximal APC concentration is motivated by concentrations of the model of Lee et al. [51], which is the basis of our signalling module (see model description in [54]). Since previous analyses have revealed that APC concentrations higher than 100 nM have only a minor impact on the downstream target gene expression [54], only cases of lower APC concentrations are considered. APC concentrations lower than 100 nM have also been measured experimentally in mammalian cells [85]. The total Wnt concentration is fixed for each cell to 1 nM since this concentration is described to induce a strong impact on mRNA levels in the model [54].

To investigate the influence of the signalling processes on target gene mRNA expression, we first consider only reactions 1-9 of the signalling module and reactions 17-18 of the target gene module (Fig. 2 b). In this setting the model species Wnt and Wnt/Frz are not explicitly included. In consequence, the concentration of the Wnt/Frz/LRP complex equals total Wnt (Additional file 1, Eq. 22). Simulations show that the APC gradient is almost completely mapped on the target gene mRNA expression profile, although with an opposite orientation, with an expression 4.5 times higher in the PC region than in the PP region (Fig. 3bA,c). Next, we additionally take processes 14 and 15 of the receptor module into account and thus explicitly include the model species Wnt and Wnt/Frz complex. The respective simulations reveal hardly any change in target gene mRNA expression (Fig. 3bB) compared to the absence of Wnt-receptor regulation (Fig. 3bA). Hence, the target gene mRNA expression is hardly affected by the addition of the Wnt receptor module.

In the next step, we investigate the impact of the autocrine effect of Dkk. We therefore include the Dkk feedback by taking additionally reactions 10-13 and 16 into account. To cover different possible conditions, we consider a weak or a strong feedback (Fig. 3bC, bD, respectively). The inclusion of the Dkk feedback reduces both the absolute target gene mRNA expression in each cell as well as the relative PC/PP expression ratio (Fig. 3c). This is caused by Dkk competing with Wnt for LRP binding, which reduces Wnt-induced signalling and thus target gene mRNA expression. Since the expression of Dkk per cell depends on the APC concentration, the impact of the Dkk feedback on target gene expression depends on the cell’s location within the APC gradient along the porto-central axis. The stronger the Dkk feedback, the more prominent the reduction of the target gene mRNA expression. In the case of the strong feedback (Fig. 3bD), absolute target gene mRNA expression is reduced by 71% PC (cell 1) and 59% PP (cell 21) compared to the combination of signalling and target gene module (Fig. 3bA). The PC/PP ratio of target gene mRNA expression is reduced from 4.5 to 3.2 (Fig. 3c).

Finally, we also include the diffusion of Dkk in the extracellular space and therefore enable paracrine regulation. The assumption of a weak diffusion rate (Fig. 3bE) results in a moderate change compared to the simulations without Dkk diffusion (Fig. 3bD) and yields a PC/PP expression ratio of 3.8 (Fig. 3c). In contrast, higher PC/PP expression ratios are obtained by fast Dkk diffusion (Fig. 3bF). In both cases the absolute target gene expression is reduced compared to the cases without Dkk regulation (Fig. 3bA, bB).

Taken together, we observe that the autocrine regulation by Dkk leads to a reduction in the target gene mRNA expression. This is accompanied by a reduction in the relative PC/PP expression levels in a liver sinusoid. These effects caused by the Dkk feedback are partly counteracted if in addition Dkk diffusion is considered. In the following we use a diffusion rate of $10\frac {\mu m^{2}}{s}$ and a Dkk translation rate (i.e., feedback strength) of 0.02 min ^−1^ as reference parameters (Fig. 3bE; Additional file 1: Table S1). This value of the diffusion coefficient corresponds to diffusion of an average protein in cytoplasm [79].

### Impact of gradient shapes on target gene mRNA expression

So far we analysed our model for a specific APC gradient along the porto-central axis and an equal Wnt level for all hepatocytes. While the existence of an APC gradient is well-established [10], the exact shape has not been reported so far. Direct spatially resolved measurements of hepatic Wnt are still missing but Wnt ligands have to be present for proper hepatic zonation [10]. Additional experiments have shown that Wnt2 and Wnt9b are produced in a more pronounced manner by endothelial cells located at the central vein than by endothelial cells located along the sinusoid [86]. This opens the possibility for higher Wnt concentrations in the PC than in the PP region. Experiments related to somitogenesis and wing development and in the intestine crypt, suggest that Wnt gradients exist in these biological contexts [87–92]. Hence, it is at least possible that a gradient of Wnt exists in the liver, too.

**Fig. 4:**
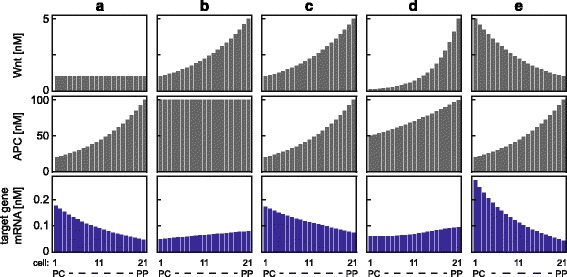
Different combinations of Wnt and APC gradients yield qualitatively different target gene mRNA expression profiles. Shown are the concentrations of total Wnt (upper row) and total APC (middle row) as well as the resulting target gene mRNA concentrations (lower row) along the porto-central axis. **a:** Constant total Wnt (1 nM) and a gradient of total APC increasing exponentially from PC (20 nM) to PP (100 nM). This represents the reference parameter set; further details are given in the Additional file 1 (Section A). **b:** Constant total APC (100 nM) and a gradient of total Wnt increasing exponentially from PC (1 nM) to PP (5 nM). **c:** Gradients of total Wnt and total APC increasing exponentially from PC (1 nM and 20 nM, respectively) to PP (5 nM and 100 nM, respectively). Both total Wnt and total APC concentrations change five-fold along the porto-central axis. **d:** Gradients of total Wnt and total APC, increasing exponentially from PC (0.1 nM and 50 nM, respectively) to PP (5 nM and 100 nM, respectively). Total Wnt concentration changes 25 times stronger than that of total APC along the porto-central axis. **e:** A gradient of total Wnt decreasing exponentially from PC (5 nM) to PP (1 nM) in combination with a gradient of total APC increasing exponentially from PC (20 nM) to PP (100 nM)

In the following, we explore to what extent the target gene mRNA expression profile is affected by different combinations of potential distributions of APC and Wnt along the porto-central axis. We start with the situation analysed in Fig. 3a, that is, an exponential gradient of total APC concentration increasing 5-fold from PC (20 nM) to PP (100 nM) and constant total Wnt concentration for each cell (1 nM) (Fig. 4 a). We compare this setting with the conditions of constant total APC concentration (100 nM) in each hepatocyte and a total Wnt concentration gradient that exponentially increases by 5-fold from PC (1 nM) to PP (5 nM) (Fig. 4 b). The simulations show that the resulting target gene mRNA expression profiles of the two conditions differ in their orientations and PC/PP ratios. The target gene mRNA expression decreases with increasing APC concentration (Fig. 4 a) but increases with increasing Wnt concentration (Fig. 4 b). While the 5-fold increase in total APC concentration from PC to PP leads to a 3.8-fold decrease in the target gene mRNA expression, the 5-fold increase in Wnt along the PC-PP axis results in a 1.6-fold increase in the target gene mRNA expression. Applying both a Wnt and an APC concentration gradient that exponentially increase 5-fold from PC to PP leads to a profile of target gene mRNA expression that decreases from PC to PP (Fig. 4 c). This is the same direction as observed in the case of constant Wnt concentration combined with the identical APC concentration gradient (Fig. 4 a). However, the PC/PP ratio of target gene mRNA expression is decreased to 2.4 (Fig. 4 c) compared to 3.8 (Fig. 4 a). The underlying reason is that the high Wnt concentration in the PP region counteracts the negative effect of high APC concentration on *β*-catenin concentration resulting in higher target gene mRNA expression in the PP region. By increasing the Wnt concentration gradient further to 50-fold from PC to PP and reducing the APC concentration gradient to 2-fold, the target gene mRNA expression profile changes its orientation (Fig. 4 d) in the same direction as observed for the combination of constant total APC concentration and an exponential total Wnt concentration gradient (Fig. 4 b). Such a dependence of the direction of the target gene mRNA expression profile on the steepness of the considered gradients does not occur if Wnt and APC concentration gradients are oriented in opposite directions (Fig. 4 e). In that case, the target gene mRNA expression profile always opposes the direction of the APC gradient independent of parametrisation and steepness of the assumed gradients (Additional file 1, Section B.2).

In summary, our simulations reveal that the APC gradient strongly affects the spatial profile of target gene mRNA expression. Additional gradients in Wnt might contribute to the particular shape of the profile. An opposite orientation of Wnt and APC gradients (Fig. 4 e) always results in target gene mRNA expression profiles that have opposite orientation with respect to the applied APC concentration gradient. In contrast, APC and Wnt concentration gradients oriented in the same direction along the porto-central axis can give rise to target gene mRNA expression profiles in the same as well as opposite direction of the applied APC concentration gradient depending on the respective steepness of the APC and Wnt concentration gradients (Fig. 4 c, d). However, this latter scenario is less likely since experiments indicate rather a higher Wnt concentration in the PC region [86]. Hence, the scenarios presented in Fig. 4 b-d are not very likely in a healthy liver and are not further investigated in the present study.

Moreover, the setting with equal APC concentration in all cells (Fig. 4 b) seems highly unlikely in a healthy liver, but might occur under abnormal circumstances, which affect APC turnover or the formation and maintenance of the APC gradient.

Both the scenarios in Fig. 4 a and in Fig. 4 e are biologically reasonable and do not contradict experimental findings regarding the presence of APC and Wnt. Less assumptions on Wnt are required in the scenario with equal Wnt concentration for each hepatocyte. Therefore, in the following analyses, we focus on the scenario presented in Figs. 4 a and 3 presuming an APC gradient increasing 5-fold from 20 nM (PC) to 100 nM (PP) in combination with a constant total Wnt concentration of 1 nM acting on each cell.

### Impact of a single mutant cell on target gene mRNA expression

#### Comparison of wild-type and mutant scenario

**Fig. 5:**
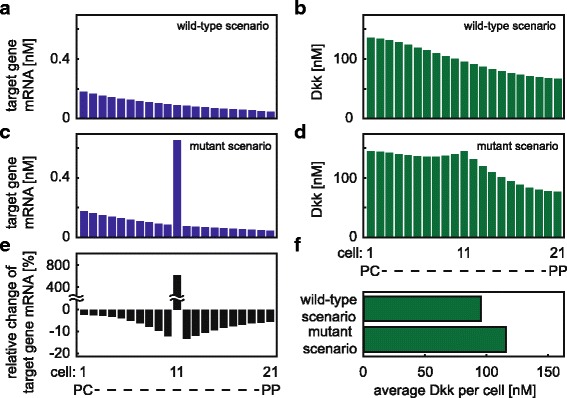
Impact of a mutant cell on target gene mRNA expression and Dkk concentration. **a, b:** Target gene mRNA expression (**a**) and Dkk concentration (**b**) profiles in the wild-type scenario. **c, d:** Target gene mRNA expression (**c**) and Dkk concentration (**d**) profiles in the presence of a cell harbouring a mutation that impedes APC-dependent *β*-catenin degradation. In the model the mutation is realised by a ten-fold decrease in the APC-dependent *β*-catenin degradation rate (*k*_3_) in the cell positioned at the centre of the porto-central axis (cell 11). **e**: Relative change of target gene mRNA expression of the mutant scenario (**c**) compared to the wild-type scenario (**a**), i.e. $\frac {[\text {target gene mRNA}]_{\text {mutant scenario}}-[\text {target gene mRNA}]_{\text {wild-type scenario}}}{[\text {target gene mRNA}]_{\text {wild-type scenario}}}$, stated in %. **f:** The average concentration of Dkk per cell is compared between wild-type (**b**) and mutant (**d**) conditions. The reference parameter set (Additional file 1: Table S1) is considered in the simulations

Aberrant activation of the Wnt/ *β*-catenin pathway is frequently observed in human cancer such as HCC [93–96]. In HCCs, mutations in *β*-catenin, Axin or APC have been detected that interfere with the degradation of *β*-catenin by the destruction complex [39–43]. Here we explore to what extent a mutation in a single hepatocyte can impair signal transduction in the surrounding wild-type cells. In the model, the mutation is implemented by decreasing the APC-dependent degradation rate of *β*-catenin (Fig. 2 b, reaction 3). This mutation is present in a single cell along the porto-central axis (referred to as mutant cell) while for all other cells the wild-type condition is considered. We compare the target gene mRNA expression of such a mutant scenario with that of the wild-type scenario, in which wild-type conditions are considered for all 21 cells. We start our investigation assuming that the mutant cell is positioned in the centre of the porto-central axis (cell 11). The mutation is realised by a 10-fold decrease in the APC-dependent *β*-catenin degradation rate (*k*_3_) compared to that in a wild-type cell. The simulations show that the target gene mRNA expression of the mutant cell is strongly increased compared to that of cell 11 in the wild-type scenario (Fig. 5 a, c). This increased expression results from the decreased APC-dependent *β*-catenin degradation under mutant condition. In addition to the increased target gene mRNA expression, the mutant cell also produces more Dkk compared to cell 11 in the wild-type scenario. Due to diffusion, Dkk is distributed along the porto-central axis resulting in increased Dkk levels for all cells in the mutant scenario compared to wild type scenario (Fig. 5 b, d, f). In the considered case, Dkk is increased on average by about 20% (Fig. 5 f). Since Dkk counteracts the pathway activation induced by Wnt, the target gene mRNA expression of the surrounding wild-type cells is decreased in the mutant scenario compared to wild-type scenario (Fig. 5 e). The decrease of the target gene mRNA expression is strongest in the cells directly adjacent to the mutant cell (cells 10 and 12) and becomes less with increasing distance from the mutant cell (Fig. 5 c). The effect is slightly asymmetric; the relative change is slightly more pronounced in cell 12 than in cell 10 (Fig. 5 e). The reason is that cells towards PC already produce more Dkk than cells towards PP due to their lower APC concentrations.

Taken together, the incorporation of a mutation leads to a strong up-regulation of target gene mRNA expression in the mutant cell. The mutant cell also produces more Dkk than any wild-type cell along the porto-central axis. By means of Dkk diffusion, the mutant cell impacts neighbouring cells in a paracrine manner, which leads to a reduction of their target gene expression. The absolute changes of target gene mRNA expression in the surrounding cells are less pronounced than that in the mutant cell itself. The strongest paracrine effect of the mutant cell, i.e., the most pronounced relative change of target gene mRNA expression in the surrounding cells, occurs in the immediate neighbour towards the PP region. We will therefore use the immediate neighbour towards the PP region as so-called readout cell in the following analyses.

#### Effect of mutant cell’s position on target gene mRNA expression

**Fig. 6:**
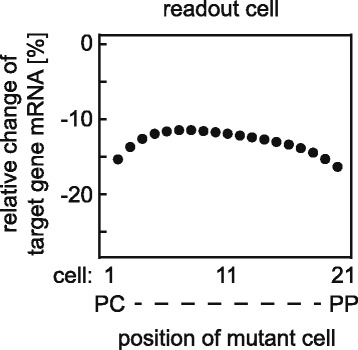
Effect of the mutant cell’s position on target gene mRNA expression. The position of the mutant cell along the porto-central axis is varied and the impact on target gene mRNA expression of the readout cell is plotted

First, we tested whether these results depend on the position of the mutant cell in the APC gradient. To this end, we studied the impact of the position of the mutant cell within the gradient on the target gene mRNA expression of the readout cell. We find that the effect on the neighbouring cell is only slightly dependent on the mutant’s location within the gradient (Fig. 6). Target gene expression in the readout cell is reduced between 12% (if the mutant cell occurs in the centre of the gradient) and 16% (if the mutation occurs in cell 2 or 20). The slightly stronger effect in the cases of mutant cell positions 2 or 20 compared to position 11 is due to the boundary condition of our diffusion model.

#### Effect of mutation strength on target gene mRNA expression

**Fig. 7:**
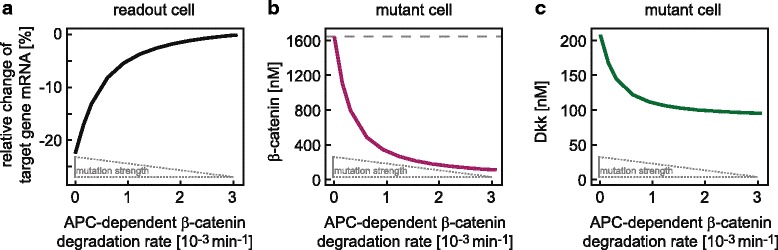
Effect of mutation strength on target gene mRNA expression and Dkk concentration. **a:** The impact on target gene mRNA expression of the readout cell is computed for different APC-dependent *β*-catenin degradation rates (*k*_3_). A decrease in the APC-dependent *β*-catenin degradation rate represents an increase in mutation strength (indicated by the grey inlet). **b:** *β*-Catenin steady state concentrations in the mutant cell are computed for different APC-dependent *β*-catenin degradation rates (*k*_3_). The dashed line indicates the maximum possible *β*-catenin concentration, which is determined by the synthesis rate (*v*_1_) and APC-independent degradation rate constant (*k*_2_). **c:** Extracellular Dkk concentration at the location of the mutant cell (cell 11) is computed for different APC-dependent *β*-catenin degradation rates (*k*_3_). All other parameters, including the diffusion coefficient D, are set to the reference parameter values (Additional file 1: Table S1) in the simulations

One might also ask how strongly the results depend on the assumed mutation strength. To simulate increasing mutation strength, the APC-dependent *β*-catenin degradation rate constant *k*_3_ of the mutant cell is reduced. The smaller this rate constant, the more Dkk is produced by the mutant cell (Fig. 7 c) and the stronger is the impact on the target gene mRNA expression in the readout cell (Fig. 7 a). In the extreme case of no APC-dependent *β*-catenin degradation (*k*_3_=0) there is a limit in the impact on target gene mRNA expression. The reason is that in this extreme case, the *β*-catenin concentration is fixed to the ratio of its synthesis rate and APC-independent degradation rate constant (reactions 1 and 2, respectively). Consequently, the Dkk concentration that can be produced by a mutant cell is limited by this maximal possible *β*-catenin concentration (Fig. 7 b, c).

#### Effect of diffusion on target gene mRNA expression

**Fig. 8:**
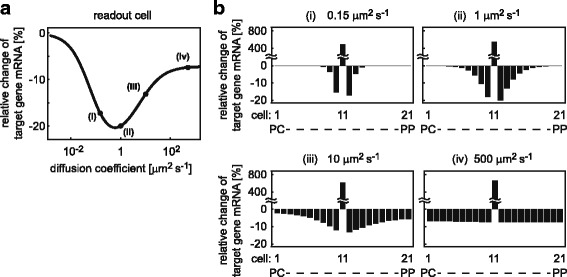
Effect of the diffusion coefficient on target gene mRNA expression. **a:** The value of the diffusion coefficient is varied over several orders of magnitude and the relative change of target gene mRNA expression in the readout cell is calculated. **b:** The influence of the diffusion coefficient on magnitude and spatial range of target gene expression is exemplarily shown for different diffusion coefficients, as indicated in (**a**): (i) *D*=0.15 $\frac {\mu m^{2}}{s}$, (ii) *D*=1 $\frac {\mu m^{2}}{s}$, (iii) *D*=10 $\frac {\mu m^{2}}{s}$ (reference parameter set), (iv) *D*=500 $\frac {\mu m^{2}}{s}$

To investigate the impact of the Dkk diffusion coefficient on the relative change in target gene mRNA expression of the readout cell, the diffusion coefficient is varied between 10^−3^ and $10^{3} \frac {\mu m^{2}}{s}$ (Fig. 8 a). Assuming a very small diffusion coefficient $\left (10^{-3}\frac {\mu m^{2}}{s}\right)$, hardly any difference in target gene mRNA expression between wild-type and mutant scenario is observed because diffusion is too weak for Dkk produced by the mutant cell to reach neighbouring cells (Fig. 8 a). The larger the diffusion coefficient becomes, the stronger is the impact on target gene mRNA expression in the readout cell until the strongest impact is observed at a diffusion coefficient of about $0.6\frac {\mu m^{2}}{s}$. Further increase in the diffusion coefficient leads to a smaller impact on the target gene mRNA expression in the direct neighbourhood but an increase of the impact on more distant cells (Fig. 8). In the case of large diffusion coefficients $\left (>10^{2}\frac {\mu m^{2}}{s}\right)$, the impact on the direct neighbourhood as well as more distant cells becomes very similar.

In summary, we showed that a mutant cell affects the target gene mRNA expression along the porto-central axis. The strength of the paracrine effect of the mutant cell on wild-type cells can be modulated by changing the mutation strength or the diffusion coefficient of Dkk. While small diffusion coefficients result in a rather local impact in the immediate neighbourhood of the mutant cell, larger diffusion coefficients reduce the local impact by distributing it across longer distances. While stronger mutations induce a stronger downregulation of target gene mRNA expression, we found the maximum possible impact to be limited. The position of the mutant along the porto-central axis has only minor impact on our results.

### Quantification of Dkk distributed by a mutant cell

So far, our analyses of the mutant scenario revealed that the mutant cell affects the target gene mRNA expression in wild-type cells but that this impact is limited and restricted to the cells in the vicinity of the mutant cell (Figs. 5 and 8). We wonder whether this restriction of the paracrine effect is due to the specific parametrisation of our model or whether this is a more general property. To address this question, we analysed the maximum impact of paracrine regulation in our model. The impact is strongly dependent on the amount of Dkk mediating the paracrine regulation, that is, Dkk which originates from the mutant cell and reaches the adjacent wild-type hepatocytes; hereafter referred to as Dkk*. To our knowledge, it is experimentally very difficult to measure extracellular Dkk directly in vivo or *in situ*. We thus aimed to derive a mathematical expression to estimate the maximum amount of Dkk* that depends on experimentally accessible quantities. Our previous analysis (Fig. 7 c) showed that Dkk of the mutant cell is maximised in the case of maximum mutation strength (*k*_3_=0). Using this assumption of maximum mutation strength, we derive an analytic equation to quantify Dkk* (Additional file 1, Section C). The equation reveals that Dkk* reaching a particular wild-type cell can be calculated from the difference of Dkk mRNA under wild-type and mutant condition at the location of the mutant cell. The feasibility of spatially resolved quantification of mRNA in liver has recently been shown [97]. Furthermore, Dkk* depends on the ratio of Dkk translation and degradation rates (*k*_12_/ *k*_13_), which can also be experimentally determined [80]. Finally, Dkk* depends on a proportion factor (*k*_*p*_), which quantifies the maximum fraction of mutant-derived Dkk reaching wild-type hepatocytes. For any wild-type cell, we computed an upper bound for this proportion factor that only depends on the cell’s distance from the mutant cell and is independent of other model parameters (Table 1). Hence, all processes that determine the maximum possible paracrine regulation can either be measured in experiments or be theoretically estimated in a parameter-independent manner.

**Table 1:** Proportion of mutant cell-produced Dkk reaching a wild-type cell. A parameter-independent upper bound for the proportion of mutant cell (cell 11) produced Dkk reaching a wild-type cell is computed depending on the distance between both cells (derived in Additional file 1, section C)

Distance to mutant cell	Parameter-independent upper bound
1 cell	17.2%
2 cells	9.0%
3 cells	6.1%
4 cells	4.9%
5 cells	4.8%

Although this analysis does not allow for direct conclusions on the maximum possible effect of a mutant cell on the gene expression in surrounding wild-type cells it demonstrates that only a small fraction of Dkk produced by the mutant cell acts via paracrine regulation on the surrounding wild-type hepatocytes.

## Discussion

The hepatocytes that adjoin liver sinusoids constitute an example in which spatial organisation is of critical importance for gene expression and cell function, and consequently for the adequate performance of the liver in the mammalian body. Factors generating the functional zonation of the liver sinusoids are concentration gradients of extracellular factors, such as oxygen, hormones and morphogens as well as intracellular components of the Wnt/ *β*-catenin signalling pathway [6–12]. Our study investigated the autocrine and paracrine influence of Dkk, an inhibitor of Wnt/ *β*-catenin signalling that is secreted by hepatocytes [25, 26, 28–34]. We investigated the impact of Dkk on the spatial expression profiles of hepatic target genes in the presence of an APC gradient and Wnt stimulus under wild-type and mutant conditions.

The results of our study can be summarised into two main points. First, the autocrine regulation by the Dkk feedback generally reduces target gene mRNA expression in wild-type hepatocytes as well as the steepness of the target gene mRNA expression profile along the porto-central axis in comparison to the simulation without Dkk feedback. This influence can be counteracted by Dkk diffusion because diffusion distributes and dilutes Dkk within the sinusoid. Second, a single cell harbouring a mutation that impedes APC-dependent *β*-catenin degradation amidst 20 wild-type hepatocytes already causes a considerable increase in the average extracellular Dkk concentration in the space of Disse. However, our simulations demonstrate that the paracrine influence of this mutation induced Dkk concentration has only limited impact on the wild-type hepatocytes, and only in the immediate vicinity of the mutant cell.

Our analysis shows that the impact of mutant cell derived Dkk on wild-type neighbours is limited on multiple levels. First, the model predicts an upper limit in the Dkk concentration that the mutant cell can produce due to the mutation that impedes the APC-dependent *β*-catenin degradation. This maximum is a consequence of the maximum possible *β*-catenin concentration, which is reached if APC-dependent degradation of *β*-catenin is completely lost. Second, the diffusion process in combination with Dkk clearance limits the amount of Dkk that eventually reaches the neighbouring wild-type hepatocytes. We quantified how much of the Dkk produced by the mutant cell actually reaches wild-type cells in the immediate neighbourhood by means of a proportion factor and calculated an upper bound. This proportion factor decreases with increasing distance of the wild-type hepatocyte from the mutant cell, illustrating the spatial limitation of the paracrine Dkk impact to the immediate vicinity of the mutant cell. Third, the autocrine Dkk feedback regulation of the hepatocytes reduces the impact of mutant cell derived Dkk on target gene mRNA expression. Dkk produced by a wild-type hepatocyte can suffice to saturate the cell’s LRP receptors. In this way, it counteracts the paracrine influence of the Dkk that is produced by the mutant cell. Finally, the signalling events of the wild-type hepatocytes limit the possible impact of LRP-bound Dkk by restricting concentrations of signal mediators such as LRP receptor, Dsh, and *β*-catenin.

Our model predicts an impact of Dkk on hepatic target gene expression in the vicinity of a mutant cell. This prediction of local impact is distinct from the results of the published Dkk diffusion model in hair follicle development [33] or in the colon [34]. These models adapt modified Turing models [98]. There, a pathway activator and inhibitor are produced by each cell and both diffuse and affect surrounding cells. This mechanism allows for far-ranging pattern formation [99]. Such patterns do not occur if only a negative feedback regulator is considered such as Dkk in our model. Since experimental observations suggest that hepatocytes do not produce Wnt [9, 86] we did not consider a positive Wnt-mediated feedback mechanism in our approach.

While we have shown that the paracrine influence of mutant cell produced Dkk is limited in magnitude and space, its impact can still be augmented in our model. An obvious approach is to assume several mutant cells since they collectively produce more Dkk than the maximal Dkk concentration that can be produced by a single mutant cell. Simulations show that additional mutant cells indeed augment the impact of a single cell (Additional file 1: Figure S2). A further possibility to increase the impact of mutant-derived Dkk is that the mutation might allow for a more efficient translation of Dkk mRNA in contrast to the surrounding wild-type cells. Under this specific assumption, the change of target gene mRNA expression in the wild-type hepatocytes could be reduced to about 50% in our model (Additional file 1: Figure S3).

It is also conceivable that larger quantities of Dkk originate from more distant sources and reach the sinusoid through the blood stream and affect Wnt/ *β*-catenin dependent target gene expression in hepatocytes. For a detailed analysis of such a scenario, an extension of our model would be necessary that takes into account processes of convection via the blood stream, transition of Dkk from blood vessels into the space of Disse as well as multi-dimensional diffusion of Dkk in that space. Such an extended model could also be used to investigate whether a local increase of Dkk in the space of Disse caused by mutant hepatocytes results in higher concentrations of Dkk in the blood.

The compelling number of published mathematical models addressing Wnt/ *β*-catenin signalling [45–47] processes demonstrates the strong demand to mechanistically understand the regulation of this pathway. To our knowledge, our model is the first that combines Wnt/ *β*-catenin signalling events and target gene expression with their regulation by feedback mechanisms and diffusion of pathway inhibitors and embeds all these processes into the particular context of liver-specific concentration gradients and mutations. This mechanistic detail of our model was essential to identify the particular processes at the multiple levels of hepatic *β*-catenin-regulated target gene expression that limit the paracrine impact of Dkk. As all models, our model is a simplification of the complex underlying biological processes. Many elementary reactions of signalling molecules have been lumped into condensed mathematical expressions of regulatory dependencies. In consequence, sequestration of signalling components into intermediate complexes are not explicitly represented. The approach is however very common and proved very useful in ODE modelling [81–83]. In the future, our model can be used as a basis to analyse the interplay of additional extra-cellular modulators of Wnt/ *β*-catenin signalling [25, 26] and their impact on the zonation of hepatic target gene expression.

## Conclusions

We present the first detailed mathematical model that describes the autocrine and paracrine regulation of hepatic Wnt/ *β*-catenin target gene expression by the secreted pathway inhibitor Dkk in a line of wild-type hepatocytes, and in the presence of cells that harbour a mutation resulting in aberrant intracellular signal transduction. Our simulations demonstrate that the presence of a single mutant cell already considerably increases Dkk levels in the space of Disse. However, the impact of these elevated Dkk levels on wild-type hepatocytes is confined in space and magnitude. The concerted action of autocrine and paracrine regulation by Dkk and Wnt/ *β*-catenin signal transduction allows wild-type hepatocytes to largely maintain their gene expression in the presence of a mutant cell.

## Additional files

Additional file 1 A: Mathematical model
B: Steady-state analysis
C: Mathematical estimation of paracrine effect
D: Supplemental figures
Figure S1: Impact of Dkk on target gene mRNA expression.
Figure S2: Effect of the number of adjoined mutant cells on target gene mRNA expression of the readout cell.
Figure S3: Effect of Dkk translation rate on target gene mRNA expression.
Table S1: Parameters of the spatial Wnt model with Dkk diffusion and feedback. (PDF 577 kb)

Additional file 2 Matlab code. (ZIP 23 kb)

## Abbreviations

APC: Adenomatous polyposis coli protein
Axin: Axis inhibition protein
CK1: Casein kinase 1
Dkk: Dickkopf
Dsh: Dishevelled
Frz: Frizzled
GSK3: Glycogen synthase kinase 3
HCC: Hepatocellular carcinoma
LRP5/6: Low-density lipoprotein (LDL) receptor-related protein 5/6
ODE: Ordinary differential equation
PC: Pericentral
PP: Periportal
sFRP: Secreted frizzled-related proteins
TCF/LEF: T-cell factor/lymphoid enhancer factor family
