# Supplementary material for: Paracrine and autocrine regulation of gene expression by Wnt-inhibitor Dickkopf in wild-type and mutant hepatocytes
Source: BMC Syst Biol. 2017 Oct 13;11:98. doi: 10.1186/s12918-017-0470-9 (PMC5640931; doi:10.1186/s12918-017-0470-9)
Supplement: Supplementary file 1 — A: Mathematical model B: Steady-state analysis C: Mathematical estimation of paracrine effect D: Supplemental figures Figure S1: Impact of Dkk on target gene mRNA expression. Figure S2: Effect of the number of adjoined mutant cells on target gene mRNA expression of the readout cell. Figure S3: Effect of Dkk translation rate on target gene mRNA expression. Table S1: Parameters of the spatial Wnt model with Dkk diffusion and feedback. (PDF 577 kb) [file 12918_2017_470_MOESM1_ESM.pdf]

Supporting Material: Paracrine and Autocrine Regulation of Gene Expression by Wnt-Inhibitor Dickkopf in Wild-type and Mutant Hepatocytes

Niklas Hartung[1,†], Uwe Benary[2,†], Jana Wolf[2], Bente Kofahl[2,3,*]

†: equal contributor
*: corresponding author
1: University of Potsdam, Institute of Mathematics, Karl-Liebknecht-Str. 24, Potsdam, Germany
2: Mathematical Modelling of Cellular Processes, Max Delbrueck Center for Molecular Medicine, Robert-Roessle-Str. 10, Berlin, Germany
3: Current address: Institute of Physics, University of Freiburg, Hermann-Herder-Str. 3, Freiburg i. Br., Germany

Contents

A Mathematical model **1**
A.1 Model equations 1
A.2 Parametrisation 3

B Steady-state analysis **5**
B.1 Uniqueness of steady-state 5
B.2 Monotonicity of gradients 5

C Mathematical estimation of paracrine effect **6**

D Supplemental figures **7**

A Mathematical model

A.1 Model equations

We describe the signalling module of our model by a published model for Wnt signalling in the liver (1). This model is given by the following system of ordinary differential equations (ODEs)

$$\frac{d[\beta\text{-catenin}]}{dt} = v_1 - v_2 - v_3 - v_4 - v_5 \tag{1}$$

$$\frac{d[\text{APC}]}{dt} = -v_4 \tag{2}$$

$$\frac{d[\text{TCF}]}{dt} = -v_5 + v_6 - v_7 \tag{3}$$

$$\frac{d[\beta\text{-catenin/TCF}]}{dt} = v_5 \tag{4}$$

$$\frac{d[\text{Dsh}_a]}{dt} = v_8 - v_9 \tag{5}$$

along with the conservation relations for total APC and total Dsh concentrations

$$[\beta\text{-catenin/APC}] + [\text{APC}] = \text{APC}^{total} \tag{6}$$

$$[\text{Dsh}_a] + [\text{Dsh}_i] = \text{Dsh}^{total} \tag{7}$$

and the rate equations (indexation corresponds to the reaction schemes in Fig. 2 B in the main text)

$$v_1 = c_1 \tag{8}$$

$$v_2 = k_2 \cdot [\beta\text{-catenin}] \tag{9}$$

$$v_3 = k_3 \cdot \frac{[\text{APC}] \cdot [\beta\text{-catenin}]}{K + [\text{Dsh}_a]} \tag{10}$$

$$v_4 = k_4 \cdot [\text{APC}] \cdot [\beta\text{-catenin}] - k_{-4} \cdot [\beta\text{-catenin/APC}] \tag{11}$$

$$v_5 = k_5 \cdot [\text{TCF}] \cdot [\beta\text{-catenin}] - k_{-5} \cdot [\beta\text{-catenin/TCF}] \tag{12}$$

$$v_6 = c_6 \tag{13}$$

$$v_7 = k_7 \cdot [\text{TCF}] \tag{14}$$

$$v_8 = k_8 \cdot [\text{Wnt/Frz/LRP}] \cdot [\text{Dsh}_i] \tag{15}$$

$$v_9 = k_9 \cdot [\text{Dsh}_a]. \tag{16}$$

To describe the receptor module, we adapt a published two-receptor model (2):

$$\frac{d[\text{Frz}]}{dt} = -v_{14} \tag{17}$$

$$\frac{d[\text{Wnt/Frz/LRP}]}{dt} = v_{15} \tag{18}$$

$$\frac{d[\text{Dkk/LRP}]}{dt} = v_{16} \tag{19}$$

with the conservation relations of total LFP, total Frz, and total Wnt concentration

$$[\text{LRP}] + [\text{Dkk/LRP}] + [\text{Wnt/Frz/LRP}] = \text{LRP}^{total} \tag{20}$$

$$[\text{Frz}] + [\text{Wnt/Frz}] + [\text{Wnt/Frz/LRP}] = \text{Frz}^{total} \tag{21}$$

$$[\text{Wnt}] + [\text{Wnt/Frz}] + [\text{Wnt/Frz/LRP}] = \text{Wnt}^{total} \tag{22}$$

and the rate equations

$$v_{14} = k_{14} \cdot [\text{Wnt}] \cdot [\text{Frz}] - k_{-14} \cdot [\text{Wnt/Frz}] \tag{23}$$

$$v_{15} = k_{15} \cdot [\text{Wnt/Frz}] \cdot [\text{LRP}] - k_{-15} \cdot [\text{Wnt/Frz/LRP}] \tag{24}$$

$$v_{16} = k_{16} \cdot [\text{Dkk}] \cdot [\text{LRP}] - k_{-16} \cdot [\text{Dkk/LRP}]. \tag{25}$$

A downstream gene expression module is added for Dkk mRNA and a generic target gene mRNA:

$$\frac{d[\text{Dkk mRNA}]}{dt} = v_{10} - v_{11} \tag{26}$$

$$\frac{d[\text{target gene mRNA}]}{dt} = v_{17} - v_{18} \tag{27}$$

with the rate equations

$$v_{10} = k_{10} \cdot [\beta\text{-catenin/TCF}] \tag{28}$$

$$v_{11} = k_{11} \cdot [\text{Dkk mRNA}] \tag{29}$$

$$v_{17} = k_{17} \cdot [\beta\text{-catenin/TCF}] \tag{30}$$

$$v_{18} = k_{18} \cdot [\text{target gene mRNA}]. \tag{31}$$

We suppose that extracellular Dkk is produced at a rate

$$v_{12} = k_{12} \cdot \frac{V_{\mathrm{Hep}}}{V_{\mathrm{Disse}}} \cdot [\mathrm{Dkk\ mRNA}] \tag{32}$$

where k_{12} is the translation rate constant and $\frac{V_{\mathrm{Hep}}}{V_{\mathrm{Disse}}}$ the ratio of intra- to extracellular volumes, i.e. the volume of one hepatocyte (V_{Hep}) and the volume of space of Disse belonging to one hepatocyte (V_{Disse}). The ratio accounts for the difference in Dkk concentrations inside hepatocytes and in the space of Disse. We also assume that Dkk is cleared from extracellular space at a rate

$$v_{13} = k_{13} \cdot [\mathrm{Dkk}]. \tag{33}$$

Furthermore, Dkk is supposed to diffuse at a rate D, yielding the following reaction-diffusion equation

$$\partial_t[\mathrm{Dkk}] = D \cdot \partial_x^2[\mathrm{Dkk}] + v_{12} - v_{13} - v_{16}, \tag{34}$$

where the porto-central axis is represented as the interval $[0, 1]$. The equation is discretised in space to contain 21 cells along the porto-central axis (hence, the discretisation step is $\Delta x = 0.05$), yielding an ODE for each cell i:

$$\frac{d[\mathrm{Dkk}]^i}{dt} = \frac{D}{\Delta x^2} \cdot \left([\mathrm{Dkk}]^{i-1} - 2 \cdot [\mathrm{Dkk}]^i + [\mathrm{Dkk}]^{i+1}\right) + v_{12} - v_{13} - v_{16}. \tag{35}$$

A Neumann boundary condition is assumed, meaning that there is no Dkk transfer out of and into the gradient through the ends of the porto-central axis.

A.2 Parametrisation

The reference parameter set is provided in Table S1. Unless explicitly stated otherwise, an exponential gradient of total APC increasing from 20 nM PC to 100 nM PP and a constant total Wnt concentration of 1 nM were assumed. The assumption of this particular shape of gradient affects results stated in absolute terms, but relative or qualitative assertions are robust to moderate changes in gradients.

For the theoretical considerations in this supplement, it is more convenient to consider the parameters $D^* = \frac{D}{\Delta x^2}$ instead of D and $k_{12}^* = k_{12} \cdot \frac{V_{\mathrm{Hep}}}{V_{\mathrm{Disse}}}$ instead of k_{12}.

Table S1: *Parameters of the spatial Wnt model with Dkk diffusion and feedback. *: Since parameters related to the reactions of the target gene module (reactions 10-13 and 17-18) are not known precisely, they were set according to reported ranges in (3). **: derived from the assumption diameter of space of Disse ≈ 2% diameter of hepatocyte from (4). †: Dkk diffusion coefficient was presumed to lie in the range of diffusion of an average protein in cytoplasm. ‡: total Frz concentrations were chosen in the range of the other species, and such that Dkk feedback had a visible effect. The values chosen for LRP and Frz are compatible with the assertion stated in (2) that cells express less Frz receptors than LRP receptors.*

Parameter name	Value	Unit	Source
c_1	0.423	$\mathrm{nM} \cdot \mathrm{min}^{-1}$	(1)
k_2	$2.57 \cdot 10^{-4}$	min^{-1}	(1)
k_3	$3.08 \cdot 10^{-3}$	min^{-1}	(1)
K	18	nM	(1)
k_4	10^5	$\mathrm{nM}^{-1} \cdot \mathrm{min}^{-1}$	(1)
k_{-4}	$1.2 \cdot 10^8$	min^{-1}	(1)
k_5	$3.33 \cdot 10^{-2}$	$\mathrm{nM}^{-1} \cdot \mathrm{min}^{-1}$	(1)
k_{-5}	1	min^{-1}	(1)
c_6	0.686	$\mathrm{nM} \cdot \mathrm{min}^{-1}$	(1)
k_7	$8.4 \cdot 10^{-2}$	min^{-1}	(1)
k_8	$8 \cdot 10^{-3}$	$\mathrm{nM}^{-1} \cdot \mathrm{min}^{-1}$	(1)
k_9	$6.7 \cdot 10^{-4}$	min^{-1}	(1)
k_{10}	$3 \cdot 10^{-3}$	min^{-1}	*
k_{11}	10^{-2}	min^{-1}	*
k_{12}	0.02	min^{-1}	*
V_{Hep}	7000	$\mu\mathrm{m}^3$	(5)
V_{Disse}	140	$\mu\mathrm{m}^3$	(4)**
k_{13}	0.1	min^{-1}	*
D	10	$\mu\mathrm{m}^2 \cdot \mathrm{s}^{-1}$	(6) †
k_{14}	0.0047	$\mathrm{nM}^{-1} \cdot \mathrm{min}^{-1}$	(2)
k_{-14}	0.0282	min^{-1}	(2)
k_{15}	1.68	$\mathrm{nM}^{-1} \cdot \mathrm{min}^{-1}$	(2)
k_{-15}	6	min^{-1}	(2)
k_{16}	0.0618	$\mathrm{nM}^{-1} \cdot \mathrm{min}^{-1}$	(2)
k_{-16}	0.0303	min^{-1}	(2)
k_{17}	$3 \cdot 10^{-3}$	min^{-1}	*
k_{18}	1	min^{-1}	*
LRP^{total}	30	nM	close to (7)
Frz^{total}	5	nM	‡
Dsh^{total}	100	nM	(1)

B Steady-state analysis

We shall find several occasions to use the following functions:

- the receptor binding function R, mapping the Dkk gradient onto a gradient in receptor-bound Wnt (i.e. Wnt/Frz/LRP),
- the signalling function S mapping gradient in receptor-bound Wnt onto a gradient in Dkk mRNA,
- and the feedback function F mapping a gradient in Dkk mRNA to Dkk.

A steady-state is a fixed point of the concatenation of these three mappings. The interaction between cells is contained within the function F. Therefore, the functions R and S are vectors of scalar functions r and s representing receptor binding and signalling of each individual cell. For a fixed set of cellular parameters, r is decreasing in Dkk (i.e., Dkk is an inhibitor of Wnt receptor binding) and increasing in Wnt^{total} (i.e., higher levels of Wnt^{total} promote Wnt receptor binding). Furthermore, s is increasing in Wnt/Frz/LRP (i.e., receptor-bound Wnt is a pathway activator) and decreasing in APC^{total} (i.e., APC is a pathway inhibitor).

B.1 Uniqueness of steady-state

To show that steady-state is unique, we take two arbitrary steady-states St_1 and St_2 (denoted by subscripts, e.g. $[\text{Dkk}]_1^i$ is the Dkk concentration of cell i in steady-state St_1) and show that they are the same. Due to the monotonicity of r and s, $[\text{Dkk}]_1^i > [\text{Dkk}]_2^i$ yields $[\text{Dkk mRNA}]_1^i < [\text{Dkk mRNA}]_2^i$, which can be represented by

$$\left([\text{Dkk}]_1^i - [\text{Dkk}]_2^i\right) \cdot \left([\text{Dkk mRNA}]_1^i - [\text{Dkk mRNA}]_2^i\right) \leqslant 0 \text{ for each cell } i \in \{1, ..., N\}. \quad (36)$$

To simplify notations, we will use the abbreviation $\overline{\text{Dkk}}^i = [\text{Dkk}]_1^i - [\text{Dkk}]_2^i$ (idem for Dkk mRNA). In steady-state, (19) entails $v_{16} = 0$, and therefore the steady-state diffusion equations are given by

$$k_{12}^* \cdot [\text{Dkk mRNA}]^i = D^* \cdot \left(- [\text{Dkk}]^{i+1} + 2[\text{Dkk}]^i - [\text{Dkk}]^{i-1}\right) + k_{13} \cdot [\text{Dkk}]^i. \quad (37)$$

Now the difference of the two steady-state equations is multiplied by $\overline{\text{Dkk}}^i$:

$$k_{12}^* \cdot \overline{\text{Dkk}}^i \cdot \overline{\text{Dkk mRNA}}^i = k_{13} \cdot \left(\overline{\text{Dkk}}^i\right)^2 + D^* \cdot \left(- \overline{\text{Dkk}}^{i-1} + 2 \cdot \overline{\text{Dkk}}^i - \overline{\text{Dkk}}^{i+1}\right) \cdot \overline{\text{Dkk}}^i. \quad (38)$$

Summing over all cells and accounting for the Neumann boundary condition, we obtain

$$\sum_{i=1}^N \underbrace{k_{12}^* \cdot \overline{\text{Dkk}}^i \cdot \overline{\text{Dkk mRNA}}^i}_{\leq 0 \text{ according to (36)}} = k_{13} \sum_{i=1}^N \left(\overline{\text{Dkk}}^i\right)^2 + D^* \sum_{i=2}^N \underbrace{\left(\left(\overline{\text{Dkk}}^{i-1}\right)^2 - 2 \cdot \overline{\text{Dkk}}^{i-1} \cdot \overline{\text{Dkk}}^i + \left(\overline{\text{Dkk}}^i\right)^2\right)}_{\geq 0 \text{ (binomial formula)}}. \quad (39)$$

Since the left-hand side of (39) is non-positive and the second right-hand term is non-negative, the first right-hand term $k_{13} \sum_{i=1}^N \left(\overline{\text{Dkk}}^i\right)^2$ must be non-positive as well, which means that $\overline{\text{Dkk}}^i = 0$ for all i.

The monotonicity of s and r also implies that if $St_1 \neq St_2$, then $\text{Dkk}_1^i \neq \text{Dkk}_2^i$ for at least one i. Therefore, the steady-state is unique.

B.2 Monotonicity of gradients

Here we consider the following scenario. Total Wnt is supposed to be non-increasing and total APC is supposed to be (strictly) increasing from PC to PP. We will show that under these assumptions, Dkk concentrations allways decrease from PC to PP.

Let us first consider the case $D^* = 0$ (no diffusion). In this case, the cells do not interact and we can pick two arbitrary cells with $\mathrm{Wnt}_1^{total} \geqslant \mathrm{Wnt}_2^{total}$ and $\mathrm{APC}_1^{total} < \mathrm{APC}_2^{total}$. Let us assume $[\mathrm{Dkk}]_1 \leqslant [\mathrm{Dkk}]_2$. The monotonicity of the receptor binding function r implies $[\mathrm{Wnt/Frz/LRP}]_1 \geqslant [\mathrm{Wnt/Frz/LRP}]_2$. Therefore, cell 1 has both a higher pathway activation rate and a lower APC-dependent pathway inhibition than cell 2, which yields $[\mathrm{Dkk\ mRNA}]_1 > [\mathrm{Dkk\ mRNA}]_2$ (monotonicity of s). As no diffusion is assumed, Dkk is proportional to Dkk mRNA, a contradiction. We conclude that $[\mathrm{Dkk}]_1 > [\mathrm{Dkk}]_2$.

Now let us suppose $D^* > 0$. We show by contradiction that Dkk is decreasing from PC to PP. Let us therefore assume that Dkk profile was not decreasing from PC to PP. Note that it has just been shown that for the same model parameters except for $D^* = 0$ the Dkk profile decrease from PC to PP. Since Dkk steady-state concentrations depend continuously on D^*, there has to be a minimal diffusion coefficient $D_0^* \in (0, D^*]$ such that Dkk is not strictly decreasing from PC to PP. Since D_0^* is the minimal value with such a property, the Dkk gradient is monotonous (same direction as for $D^* = 0$), but not strictly: there necessarily are at least two adjacent cells $i, i+1$ with equal Dkk concentrations (anything else would contradict the minimality of D_0^*).

We now look at the steady-state equation (37) of Dkk diffusion at cell i:

$$0 = D^* \cdot ([\mathrm{Dkk}]^{i+1} - 2[\mathrm{Dkk}]^i + [\mathrm{Dkk}]^{i-1}) - k_{13} \cdot [\mathrm{Dkk}]^i + k_{12}^* \cdot [\mathrm{Dkk\ mRNA}]^i. \tag{40}$$

As $[\mathrm{Dkk}]^{i-1} \geqslant [\mathrm{Dkk}]^i = [\mathrm{Dkk}]^{i+1} \geqslant [\mathrm{Dkk}]^{i+2}$, this yields $k_{13} \cdot [\mathrm{Dkk}]^i \geqslant k_{12}^* \cdot [\mathrm{Dkk\ mRNA}]^i$ and $k_{13} \cdot [\mathrm{Dkk}]^{i+1} \leqslant k_{12}^* \cdot [\mathrm{Dkk\ mRNA}]^{i+1}$ and thus, $[\mathrm{Dkk\ mRNA}]^i \leqslant [\mathrm{Dkk\ mRNA}]^{i+1}$. However, the Dkk concentrations at the receptor level are the same, and therefore $[\mathrm{Wnt/Frz/LRP}]_1 \geqslant [\mathrm{Wnt/Frz/LRP}]_2$. Using that $[\mathrm{APC}]^i < [\mathrm{APC}]^{i+1}$, monotonicity of the signalling function s yields $[\mathrm{Dkk\ mRNA}]^i > [\mathrm{Dkk\ mRNA}]^{i+1}$, a contradiction. Thus, there cannot be any diffusion coefficient such that the Dkk monotonicity is lost.

C Mathematical estimation of paracrine effect

The amount of additional Dkk at a wild-type cell originating from a mutant (denoted by Dkk^{Add} here and by Dkk* in the main text) depends on Dkk translation and degradation rates, is modulated by diffusion and regulated through the Dkk feedback. The aim of this section is to analyse this complex interplay mathematically.

We suggest estimating Dkk^{Add} in an ansatz that retains the mutant cell's impact on wild-type cells, while being simple enough for analytical tractability. This approach is based on two assumptions.

First, we assume that the mutant cell completely loses its capacity of APC-dependent β-catenin degradation ($k_3 = 0$). This assumption results in a strong impact of the mutant cell on target gene mRNA expression of the wild-type cells (main text Fig. 6B). It furthermore interrupts the mutant cell's Dkk feedback regulation by eliminating the regulatory influence of receptor processes on β-catenin concentration in the mutant cell.

Second, we describe the mutant scenario as a combination of the wild-type scenario and a hypothetical scenario. In the hypothetical scenario Dkk is only produced by the mutant cell (cell 11); all wild-type cells do not produce Dkk. Such a hypothetical scenario is useful to describe the additional Dkk, which originates from the APC mutation in addition to the Dkk produced under wild-type condition, and how this additional Dkk is distributed among all cells. That is, the Dkk of the hypothetical scenario (Dkk^{Hyp}) approximates the additional Dkk (Dkk^{Add}). Dkk^{Hyp} is calculated by solving

$$0 = D^* \big([Dkk]_{i-1}^{Hyp} - 2 \cdot [Dkk]_i^{Hyp} + [Dkk]_{i+1}^{Hyp}\big) + k_{12}^* \cdot [Dkk\ mRNA]_i^{Hyp} - k_{13} \cdot [Dkk]_i^{Hyp}, \tag{41}$$

with

$$[Dkk\ mRNA]_i^{Hyp} = \begin{cases} \big([Dkk\ mRNA]_{11}^{Mut} - [Dkk\ mRNA]_{11}^{WT}\big) & \text{if } i = 11, \\ 0 & \text{otherwise.} \end{cases} \tag{42}$$

The Dkk^{Hyp} concentration is larger than Dkk^{Add} concentration for all cells since the regulation via negative Dkk feedback in wild-type cells is neglected in the hypothetical scenario.

The advantage of bounding Dkk^{Add} by Dkk^{Hyp} is that the latter quantity is mathematically traceable, as shown in the following.

Solving the equation system (41)-(42) for Dkk^{Hyp} yields

$$\text{Dkk}^{Hyp} = k_p \cdot \frac{k_{12}^*}{k_{13}} \cdot \left([\text{Dkk mRNA}]_{11}^{Mut} - [\text{Dkk mRNA}]_{11}^{WT}\right) \tag{43}$$

with

$$k_p = A^{-1}_{\frac{D^*}{k_{13}}} E_{11}, \tag{44}$$

where

$$A_s = \begin{pmatrix} s+1 & -s & 0 & \dots & 0 \\ -s & 2s+1 & -s & 0 & \vdots \\ 0 & \ddots & \ddots & \ddots & 0 \\ \vdots & 0 & -s & 2s+1 & -s \\ 0 & \dots & 0 & -s & s+1 \end{pmatrix} \tag{45}$$

and $E_{11}(x) = \delta_{x,11}$ is the 11th unitary vector. The proportion factor k_p is a vector (i.e. it can be computed for any wild-type cell); due to the symmetry of the porto-central axis in the hypothetical scenario, k_p only depends on the distance between that cell and the mutant cell.

As can be seen from Eq. (44), the proportion factor only depends on the diffusion-to-degradation ratio. For each distance from the mutant, a parameter-independent upper bound has been determined by maximising the proportion factor numerically over the $\frac{D^*}{k_{13}}$ ratio. This upper bound is listed in Table 1 of the main text.

In this way, Dkk^{Add} can be estimated from the parameter-independent upper bound for k_p, the Dkk translation-to-degradation ratio, and the excess Dkk mRNA in the mutant.

D Supplemental figures

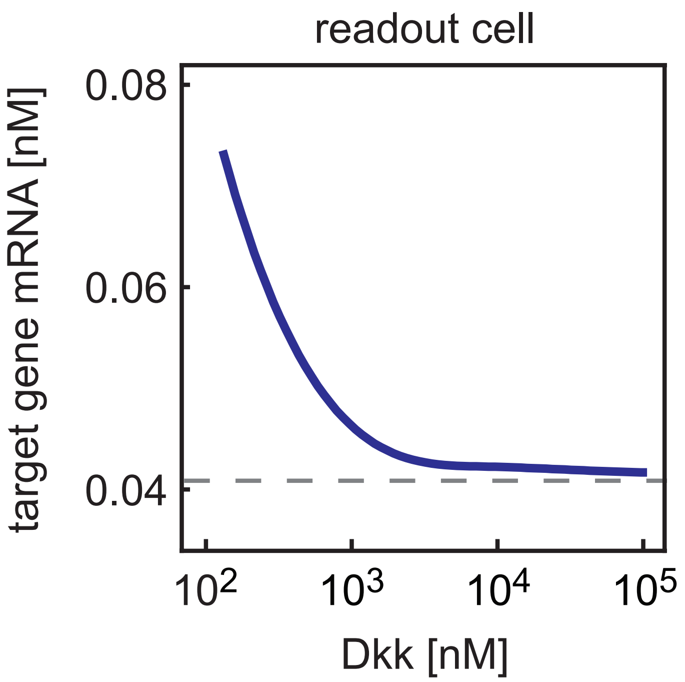

Figure S1: *Impact of Dkk on target gene mRNA expression. Target gene mRNA expression of the readout cell (cell 12) is calculated for Dkk concentrations at the readout cell ranging from 10^2 to 10^5 nM.*

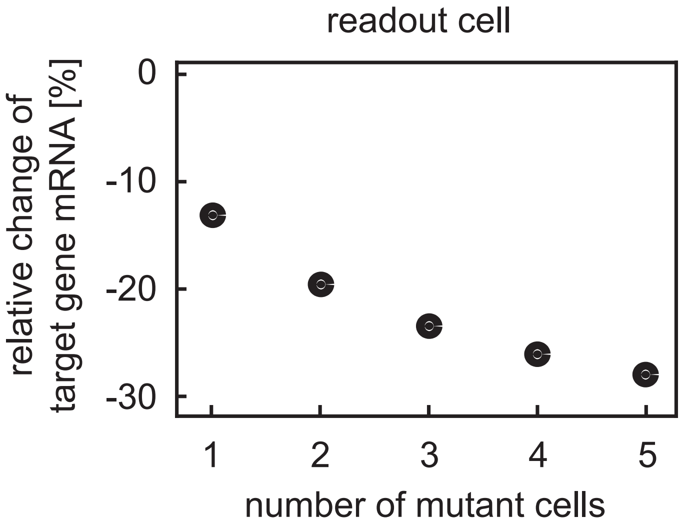

Figure S2: *Effect of the number of adjoined mutant cells on target gene mRNA expression of the readout cell (cell 12). The number of adjoined mutant cells is increased from one to five and the impact on target gene mRNA expression of the readout cell is calculated. We observe a stronger impact on the target gene mRNA expression with increasing number of adjoined mutant cells.*

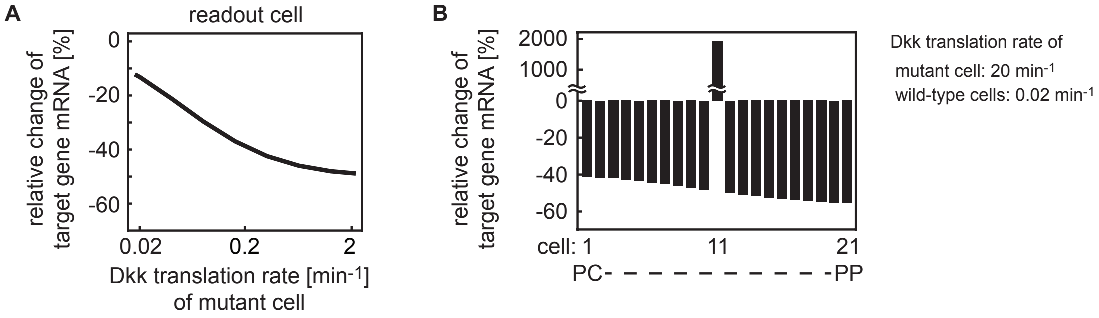

Figure S3: *Effect of Dkk translation rate on target gene mRNA expression. The Dkk translation rate of the mutant cell is increased; the Dkk translation rate of all wild-type cells remains unchanged.* **A:** *The impact on target gene mRNA expression of the readout cell is calculated.* **B:** *The impact on all cells along the porto-central axis is calculated for a large value of Dkk translation rate of the mutant cell (k_{12} =20 min^{-1}). This value is sufficiently large to yield the maximal possible impact. The analysis demonstrates that a larger value of the Dkk translation rate of the mutant cell can augment the impact on target gene mRNA expression.*

References

[1] Benary, U., Kofahl, B., Hecht, A., Wolf, J.: Modeling Wnt/beta-Catenin Target Gene Expression in APC and Wnt Gradients Under Wild Type and Mutant Conditions. Front. Physiol. **4**, 21 (2013)

[2] Kogan, Y., Halevi-Tobias, K.E., Hochman, G., Baczmanska, A.K., Leyns, L., Agur, Z.: A new validated mathematical model of the Wnt signalling pathway predicts effective combinational therapy by sFRP and Dkk. Biochem. J. **444**(1), 115–125 (2012)

[3] Schwanhäusser, B., Busse, D., Li, N., Dittmar, G., Schuchhardt, J., Wolf, J., Chen, W., Selbach, M.: Global quantification of mammalian gene expression control. Nature **473**(7347), 337–342 (2011)

[4] Kuntz, E., Kuntz, H.-D.: Hepatology: Textbook and Atlas. Springer, Berlin/Heidelberg (2008)

[5] Plettenberg, S., Weiss, E.C., Lemor, R., Wehner, F.: Subunits alpha, beta and gamma of the epithelial Na+ channel (ENaC) are functionally related to the hypertonicity-induced cation channel (HICC) in rat hepatocytes. Pflugers Arch. **455**(6), 1089–1095 (2008)

[6] Moran, U., Phillips, R., Milo, R.: SnapShot: Key Numbers in Biology. Cell **141**(7), 1262–12621 (2010). doi:10.1016/j.cell.2010.06.019

[7] Pedersen, L., Jensen, M.H., Krishna, S.: Dickkopf1–a new player in modelling the Wnt pathway. PLoS ONE **6**(10), 25550 (2011)
